# Draft Genome Sequence of *Magnetospirillum* sp. Strain 15-1, a Denitrifying Toluene Degrader Isolated from a Planted Fixed-Bed Reactor

**DOI:** 10.1128/genomeA.00764-17

**Published:** 2017-08-10

**Authors:** Ingrid Meyer-Cifuentes, Stefan Fiedler, Jochen A. Müller, Uwe Kappelmeyer, Ines Mäusezahl, Hermann J. Heipieper

**Affiliations:** a Helmholtz Centre for Environmental Research–UFZ, Department of Environmental Biotechnology, Leipzig, Germany; b Robert Koch Institute, Division of Nosocomial Pathogens and Antibiotic Resistances, National Reference Centre for Staphylococci and Enterococci, Department of Infectious Diseases, Wernigerode, Germany

## Abstract

Here, we report the draft genome sequence of *Magnetospirillum* sp. 15-1. This strain was isolated from a planted fixed-bed reactor based on its ability to degrade toluene under anaerobic conditions. The genome assembly consists of 5.4 Mb in 28 contigs and 5,095 coding sequences containing the genes involved in anaerobic toluene degradation.

## GENOME ANNOUNCEMENT

Anaerobic degradation of toluene has been investigated most intensively among *Betaproteobacteria* strains, represented by *Thauera aromatica* and *Azoarcus* sp. (1–3). Within the *Alphaproteobacteria* group, there are three strains that have been identified as toluene degraders, *Blastochloris sulfoviridis* (4), *Magnetospirillum* sp. TS-6 (5), and *Magnetospirillum* sp. 15-1 (6).

Here, we present the draft genome sequence of *Magnetospirillum* sp.15-1, a nonmagnetic strain isolated from a planted fixed-bed reactor (PFR) continuously fed with toluene (6). Sequencing was carried out using the 454-GS Junior system (Roche Applied Science) and MiSeq instrument (Illumina). Illumina libraries were paired-end sequenced with a maximum 300-bp read length (MiSeq reagent kit version 3, 600 cycles, Illumina). Together, sequencing resulted in 1,674,000 reads. *De novo* assembly was conducted with the A5-miseq pipeline (7) and Geneious assembler (http://www.geneious.com) (8), generating 527 contigs initially. Contigs shorter than 400 bp with a coverage percentage below 20, an aberrant GC content below 50%, and a low *Q* score (9) were not considered further. The relative proportion of omitted nucleotides was 1.2%. Five gaps were closed by PCR, resulting in 28 final contigs. The draft genome sequence of *Magnetospirillum* sp. 15-1 consists of 5,422,505 bp with an average G+C content of 65.6%. The genome contains 6 rRNAs, 49 tRNAs, and 5,095 coding sequences (CDSs) (coding percentage 98.82%). The assembled sequences were functionally annotated using the RAST online service (10) and IMG (11). Of 5,095 CDSs, 62.61% were assigned to at least one COG group.

Magnetic strains of *Magnetospirillum* contain a large magnetosome genomic island that harbors a high concentration of insertion sequences and is flanked by repetitive elements (12, 13). This island is absent in strain 15-1. Phylogenetic analyses based on comparisons of average nucleotide identities and 16S rRNA gene alignment showed that strain 15-1 is related more closely to the magnetosome-producing strains *M. magneticum* AMB-1 (87%) (14), *M. magnetotacticum* MS-1 (86%) (15), *Magnetospirillum* sp. XM-1 (16) (88%), *Magnetospirillum* sp. SO-1 (89%) (17), and *M. gryphiswaldense* sp. MSR-1 (76%) (18) than to the nonmagnetic *M. bellicus* VDY (77%) (19) and *Magnetospirillum* sp. WD (77%) (20). This finding suggests that the absence of the magnetosome island in the *Magnetospirillum* sp. 15-1 genome is due to a secondary loss of function.

The genes for degradation of toluene under anaerobic conditions were found to be organized in three operons on the chromosome (*bss*, *bbs*, and *bam*), as observed for *Thauera aromatica* K172 (21). Homology searches of the *bss* operon of strain 15-1 against other anaerobic toluene degraders were performed through BLAST (https://blast.ncbi.nlm.nih.gov/Blast.cgi). Results showed similarities of 99% to *Magnetospirillum* sp. TS-6 and 80% to both *T. aromatica* K172 (22) and *Azoarcus* sp. (23). Analysis of xenobiotic degradation pathways through the KEGG pipeline to sequenced *Magnetospirillum* strains (excluding strain 15-1) showed that none of these strains contain genes related to aromatic compound degradation pathways.

### Accession number(s).

This whole-genome shotgun project has been deposited in the European Nucleotide Archive (ENA) under the accession no. FXXN01000001 to FXXN01000028. The versions described in this paper are the first versions.
